# Stabilization of a G-Quadruplex from Unfolding by Replication Protein A Using Potassium and the Porphyrin TMPyP4

**DOI:** 10.4061/2011/529828

**Published:** 2011-06-16

**Authors:** Aishwarya Prakash, Fabien Kieken, Luis A. Marky, Gloria E. O. Borgstahl

**Affiliations:** ^1^The Eppley Institute for Research in Cancer and Allied Diseases, University of Nebraska Medical Center, 987696 Nebraska Medical Center, Omaha, NE 68198-7696, USA; ^2^Department of Biochemistry and Molecular Biology, University of Nebraska Medical Center, Omaha, NE 68198-5870, USA; ^23^Department of Pharmaceutical Sciences, College of Pharmacy, University of Nebraska Medical Center, Omaha, NE 68198-6025, USA

## Abstract

Replication protein A (RPA) plays an essential role in DNA replication by binding and unfolding non-canonical single-stranded DNA (ssDNA) structures. Of the six RPA ssDNA binding domains (labeled A-F), RPA-CDE selectively binds a G-quadruplex forming sequence (5′-TAGGGGAAGGGTTGGAGTGGGTT-3′ called Gq23). In K^+^, Gq23 forms a mixed parallel/antiparallel conformation, and in Na^+^ Gq23 has a less stable (*T*_*M*_ lowered by *∼20*°C), antiparallel conformation. Gq23 is intramolecular and 1D NMR confirms a stable G-quadruplex structure in K^+^. Full-length RPA and RPA-CDE-core can bind and unfold the Na^+^ form of Gq23 very efficiently, but complete unfolding is not observed with the K^+^ form. Studies with G-quadruplex ligands, indicate that TMPyP4 has a thermal stabilization effect on Gq23 in K^+^, and inhibits complete unfolding by RPA and RPA-CDE-core. Overall these data indicate that G-quadruplexes present a unique problem for RPA to unfold and ligands, such as TMPyP4, could possibly hinder DNA replication by blocking unfolding by RPA.

## 1. Introduction

The observation that G-rich DNA can form a myriad of secondary structures was made in the 1960s, however, it was not until the late 1980s that three seminal papers described the in vitro formation of G-quadruplexes [1–3]. Certain G-rich sequences can fold into secondary structures composed of planar G-quartets that are stabilized by eight hydrogen bonds between the “Watson-Crick” face of one guanine base and the “Hoogsteen” face of another guanine base [4]. The stacking of two or more G-quartets forms what is called a G-quadruplex. The formation of G-quadruplexes requires the presence of monovalent cations like K^+^ and Na^+^ between the G-quartet planes. G-quadruplexes can fold into various conformations where the strands are oriented in either a parallel or antiparallel orientation and can fold either intra- or intermolecularly. 

The study of G-quadruplex structures is gaining more interest in the field of human cancers as their in vivo presence becomes increasingly more evident [5, 6]. DNA sequences with the propensity to form G-quadruplex structures are found throughout the human genome and are enriched in the promoter regions of proto-oncogenes such as *c-MYC*, *c-KIT*, *BCL-2*, *VEGF*, and *RET* [7, 8]. This list also includes hTERT and insulin promoters [9, 10]. At telomeres, the G-rich overhangs in ssDNA regions form G-quadruplex structures in vitro, and in *Stylonychia lemnae* these structures were observed in vivo [11]. Agents that stabilize G-quadruplex structures have the potential to interfere with transcription of oncogenes, prevent telomere elongation by telomerase, and disrupt the replication of G-rich sequences. Therefore, G-quadruplexes serve as potential targets for drug design [12, 13]. 

There are two main categories of G-quadruplex ligands (1) telomestatin- and amide-based macrocyclic ligands, and (2) porphyrin and related pyrrole- and isoindole-based macrocyclic ligands [8]. Here, the effects of the porphyrin* meso*-5,10,15,20-Tetrakis-(N-methyl-4-pyridyl)porphine (TMPyP4) and telomestatin, a natural product from *Streptomyces anulatus* [14, 15] are studied. TMPyP4 and telomestatin display selectivity for G-quadruplex DNA over duplex DNA reinforcing the ability of these compounds as potential candidates for cancer therapeutic agents [16, 17]. TMPyP4 binding to the G-rich element upstream of the cMyc promoter inhibits transcription [18]. Telomestatin effectively inhibits the binding of telomere-associated proteins like telomerase, POT1 and TRF2, thereby causing an uncapping of telomeres leading to activation of a DNA damage response and induction of apoptosis [19, 20]. At subtoxic concentration, telomestatin can inhibit telomerase and cause a shortening of telomeres that can result in the induction of senescence or crisis [21, 22]. TMPyP4 has at least two distinctly different binding modes [17, 23]. Crystallographic and NMR studies show TMPyP4 binding to the side and stacked on the top of G-quadruplexes suggesting a mechanism for stabilization and inhibition of protein binding [17, 23]. Unfortunately, there are no available structures for telomestatin binding; however, using simulated annealing docking, 2 telomestatin moieties were found to bind to a 24-mer telomeric G-quadruplex [14]. In this study, the effect of TMPyP4 and telomestatin on the unfolding of G-quadruplexes by replication protein A (RPA) is explored. 

Protein binding to G-quadruplexes can either stabilize the G-quadruplex fold as seen in *Oxytricha nova* telomere-end binding protein [24–26] or unfold and destabilize the structure as observed in the case of DNA helicases and RPA [26–28]. The RPA heterotrimer is the primary, eukaryotic, ssDNA binding protein that is involved in the 3 R's of eukaryotic DNA metabolism including replication, recombination, and repair [29–31]. RPA's subunits, RPA1, RPA2, and RPA3 (70, 32, and 14 kDa, resp.), are divided into six structurally similar oligonucleotide binding (OB) folds (named A-F), five of which have previously been shown to possess DNA binding activity (A-E) [32, 33]. RPA binds ssDNA with high affinity (*K*_*a*_ ~ 10^9^ − 10^11^ M^−1^) and binds polypyrimidine sequences with higher affinity than polypurine sequences [29, 34]. Recently, RPA has been shown to bind G-rich DNA. Experiments employing fluorescence resonance energy transfer (FRET) assays indicate that RPA unfolds telomeric G-quadruplexes and no detectable difference was seen if the ion was K^+^ or Na^+^ [35]. In separate studies in the presence of K^+^ ions, using circular dichroism (CD), RPA was shown to bind and melt nontelomeric intramolecular G-quadruplexes [36], and using electrophoretic mobility shift assays (EMSAs) RPA could not melt an intermolecular four-stranded G-quadruplex [37]. In this study, the effect of Na^+^ versus K^+^ ions on G-quadruplex stability against RPA unfolding is assessed using full-length RPA as well as a deletion mutant of RPA (RPA-CDE-core) that contains the OB-folds required for specific G-quadruplex binding. 

In a previous study, systematic evolution of ligands by exponential enrichment (SELEX) was used to find RPA's DNA sequence preferences [38]. SELEX results with RPA-CDE indicate preferential binding to a G-rich sequence called Gq23 that forms a G-quadruplex. Fluorescence polarization (FP) binding experiments indicated that the RPA-CDE differs from the other OB-folds in that they selectively bind G-rich DNA and cannot unfold a G-quadruplex structure. Here, the effects of K^+^ and Na^+^ ions on G-quadruplex structure and stability, NMR structural experiments, the effect of the G-quadruplex ligands TMPyP4 and telomestatin on the stability of Gq23, and their ability to inhibit unfolding by RPA and RPA-CDE-core are evaluated. Dramatic differences in G-quadruplex stabilization are revealed under conditions containing K^+^ ions and TMPyP4.

## 2. Materials and Methods

### 2.1. DNA and G-Quadruplex Ligands

A G-quadruplex with the following sequence 5′-TAGGGGAAGGGTTGGAGTGGGTT-3′ named Gq23 was synthesized by the University of Nebraska Medical Center DNA synthesis core facility and desalted prior to use. The calculated extinction coefficient (*ε*) for Gq23 was 234.6 L/(mmol*cm) and was calculated using methods previously described [39]. The porphyrin TMPyP4 (Calbiochem) and telomestatin (generously provided by Dr. Shin-ya, University of Tokyo, Japan [40]) were used as small-molecule G-quadruplex ligands.

### 2.2. RPA Constructs and Purification Scheme

Plasmid for full length human RPA was obtained from Dr. Marc Wold, University of Iowa. Plasmid for RPA-CDE-core was obtained from Dr. Walter Chazin, Vanderbilt University. Overexpression in bacteria followed standard procedures. The purification scheme of RPA followed previous protocols where the proteins were purified by fractionation over Affi-gel Blue, Hydroxyapatite, and Mono-Q columns [41, 42]. RPA-CDE-core contained a thrombin-cleavable, N-terminal His-tags and was purified as described [43] using Nickel column chromatography, tag cleavage, and Mono-Q anion exchange chromatography. Proteins were concentrated by ultrafiltration and concentrations were determined using the absorbance at 280 nm.

### 2.3. Circular Dichroism (CD) Experiments

An AVIV CD spectrometer model 202SF equipped with Peltier temperature control was used. Spectra on the DNA alone, DNA : ligand, DNA : protein, and [DNA : ligand] : protein titrations were measured in a 1 cm quartz cuvette in buffer containing 25 mM Tris pH 7.5, 2 mM MgCl_2_, 0.5% inositol, 1 mM DTT, and either 100 mM NaCl or 100 mM KCl at 25°C (or at the specified temperature). For the titration experiments, protein : DNA molar ratios from 0 to 5 were used. The concentration of the oligonucleotide used was 1.4 *μ*M in all experiments and the CD data obtained were plotted using OriginV software. All spectra were averages of 3 scans. For melting curves, the wavelength was held constant at either 260 or 292 nm and the temperature was varied between 2 and 100°C. Melting temperature (*T_M_*) values were obtained from van't Hoff analysis of UV-melting curves using procedures previously described [44]. 

### 2.4. UV Melting Curves and Differential Scanning Calorimetry (DSC)

The transition molecularity of the unfolding of a particular complex was obtained by monitoring the *T_M_* as a function of the strand concentration. For this, a thermoelectrically controlled Aviv Spectrophotometer (Model 14-DS) was used. To obtain a concentration-dependence curve, quartz cuvettes with different path lengths 0.1, 0.2, 0.5, and 1 cm were used to vary the concentration of the oligonucleotide. A plot of the *T_M_* versus the natural log (ln) of the concentration was plotted. All UV melts were performed in a buffer containing 10 mM HEPES at pH 7.4, 140 mM CH_3_COOK, 1.5 mM MgCl_2_, and 1 mM DTT, and plots were generated using Microsoft Excel and Microcal Origin 5.0 software. A *T_M_* value was also obtained using a VP-DSC from Microcal (Northampton, MA) and by plotting the apparent change in the heat capacity as a function of temperature. The DSC *T_M_* value is measured at the peak of the resultant bell-shaped curve.

### 2.5. NMR Experiments

For NMR spectra, 1 mM of the oligonucleotide was resuspended in a buffer containing 20 mM deuterated Tris pH 6.5, and 100 mM KCl in 10% D_2_O. The samples were heated to 90°C and cooled slowly to room temperature prior to NMR analysis. ^1^H NMR spectra were collected using a Varian INOVA 600 instrument with a cryoprobe, in water with 10% D_2_O, and with Watergate water suppression techniques at 7°C. ^31^P spectra were performed at 25°C using a UNITY 500 equipped with a broad-band probe using the same sample with composite pulse proton decoupling referenced to an external standard of 85% H_3_PO_4_. NMR data were processed with MestReNova software (Mestrelab S.L., Spain).

## 3. Results

### 3.1. Cation Effects on G-Quadruplex Structure and Stability

**Figure 1:**
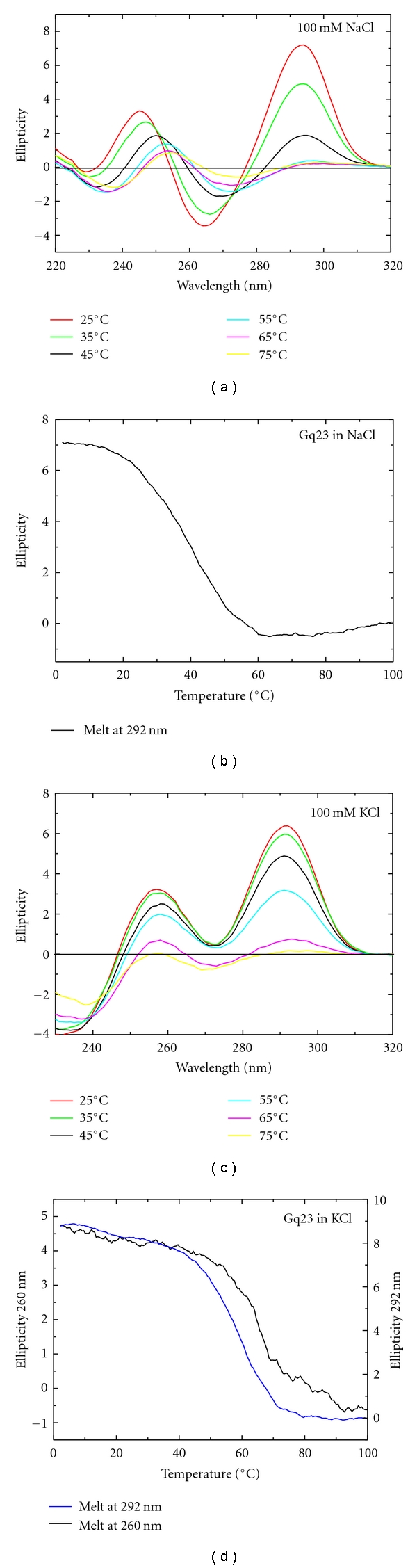
The effect of monovalent cations on Gq23 structure and thermal stability. (a) CD spectra at varying temperatures of Gq23 in a buffer containing 100 mM NaCl. (b) Gq23 melt at 292 nm in 100 mM NaCl. (c) Spectra of Gq23 in a buffer containing 100 mM KCl recorded at varying temperatures from 25°C to 75°C. (d) Gq23 melt at both 292 nm and 260 nm since both peaks were seen at both wavelengths throughout the temperature range.

RPA-CDE binds preferentially to a 23-nt G-rich sequence, called Gq23, which forms a G-quadruplex in vitro [38]. Circular dichroism (CD) data reveal that Gq23 forms a G-quadruplex in the presence of 100 mM Na^+^ or K^+^ ions. In the presence of Na^+^ ions an antiparallel G-quadruplex with a characteristic peak at 292 nm is observed (Figure 1(a)). Upon increasing the temperature to 75°C, the magnitude of the peak at 292 nm diminishes as the G-quadruplex unfolds. A melting curve monitored at a constant wavelength of 292 nm revealed a melting temperature (*T_M_*) of 40°C with a calculated van't Hoff enthalpy (ΔH_VH_) of 23 kcal (Figure 1(b), Table 1 (line 1)). There is a bathochromic shift of the 260 nm peak and it appears that the Na^+^ form of Gq23 goes through a random coil state as it unfolds. In the presence of K^+^ ions the G-quadruplex has both parallel and antiparallel characteristics as indicated by peaks at both 260 and 292 nm (Figure 1(c)). Melting curves in the presence of K^+^ ions were monitored at both wavelengths and revealed a *T_M_* of 66°C and 57°C, respectively (Figure 1(d), Table 1 (line 2)) with ΔH_VH_ values of 32 and 35 kcal. The higher *T_M_* and ΔH_VH_ values in the presence of K^+^ reveal that this G-quadruplex is more stable under these conditions.

### 3.2. Molecularity of the K^+^ G-Quadruplex

**Figure 2:**
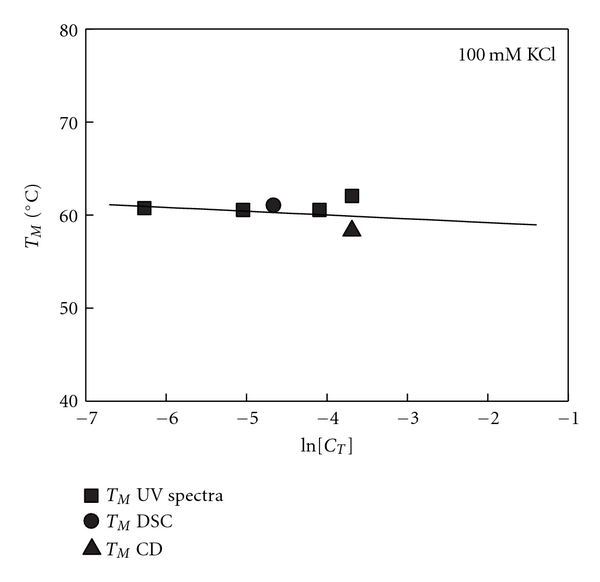
Melting temperature dependence on concentration of oligonucleotide. A linear dependence of *T_M_* with strand concentration demonstrates that the G-quadruplex formed is intramolecular. Filled squares are *T_M_* from UV melting curves, filled circle is *T_M_* from DSC and filled triangle is *T_M_* from CD.

To obtain the molecularity of the G-quadruplex that forms in the presence of K^+^ ions, UV-melting curves were obtained as a function of strand concentration. DNA bases absorb light in the far-region of the UV spectrum and the absorbance is monitored at 260, 275, or 292 nm depending on the sequence used. For the G-quadruplex DNA studied here, a hypochromic effect is observed at 292 nm and thus all the melting curves were performed at this wavelength. UV-melting curves at four concentrations were monitored at 292 nm and reveal that the unfolding transition of Gq23 is monophasic. From each melting curve, the *T_M_* values were obtained. Over the 10-fold range in strand concentration the *T_M_* remained constant, indicating that Gq23 forms an intramolecular G-quadruplex in these reaction conditions (Figure 2). Using DSC analysis, the apparent molar heat capacity of Gq23 was measured as a function of temperature. Shape analysis of the curve obtained reveals that Gq23 has a typical bell-shaped transition indicated by a single peak (data not shown). The *T_M_* is obtained from the peak of the calorimetric curve which was measured to be 61°C (Figure 2). The ratio of the van't Hoff enthalpy and the calorimetric enthalpy revealed a value approximately equal to 1 indicating a two-state transition. This *T_M_* value agrees well with those measured by CD and confirms that the G-quadruplex is unimolecular.

### 3.3. Confirmation of G-Quadruplex Structure by NMR

**Figure 3:**
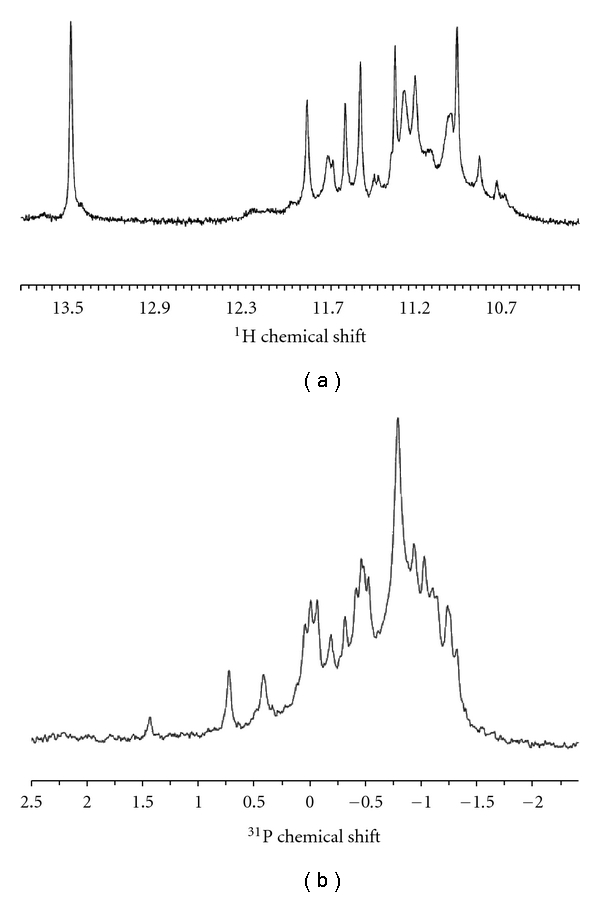
NMR analysis of Gq23 structure. (a) The imino proton region of 1D ^1^H NMR spectrum of Gq23. (b) 1D proton-decoupled ^31^P NMR spectrum of Gq23.

^1^H NMR spectra were recorded in the presence of K^+^ ions. The spectrum showed that the resonances of DNA imino protons are mainly confined between 10 and 12 p.p.m in K^+^ ions (Figure 3(a)). A peak for each guanine imino proton with a chemical shift between 10 and 12 p.p.m. is characteristic of the formation of a G-quadruplex in solution [45]. The formation of the G-quadruplex in K^+^ ions was also confirmed with a ^31^P NMR spectrum (Figure 3(b)). ^31^P resonances are spread over a 2.5 p.p.m. range indicating that the phosphodiester backbone is not uniform along the DNA, which is another characteristic of G-quadruplex formation [45]. These NMR data confirm the interpretation of the CD spectra (Figure 1) as a G-quadruplex fold that is stabilized in the presence of K^+^ ions. In the presence of Na^+^ ions, the ^1^H NMR spectrum was of poor quality and showed that the imino proton resonances were unresolved (data not shown). This indicates that the Na^+^ form of the Gq23 G-quadruplex was less stable than the K^+^ form.

### 3.4. Stabilizing Effect of K^+^ against RPA Unfolding

**Figure 4:**
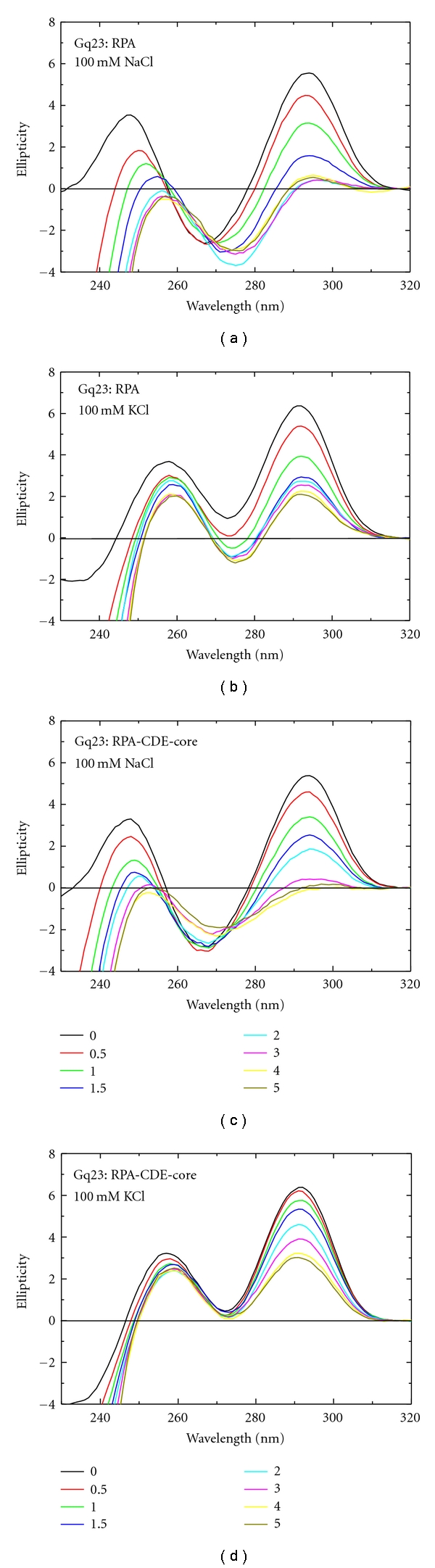
Effect of addition of RPA and RPA-CDE-core on Gq23 in the presence of Na^+^ or K^+^ ions. Addition of increasing molar ratios of RPA to Gq23 at (a) 100 mM NaCl and (b) 100 mM KCl. Addition of increasing molar ratios of RPA-CDE-core to Gq23 at (c) 100 mM NaCl and (d) 100 mM KCl.

The effect of full-length RPA, and the deletion mutant RPA-CDE-core that bounds preferentially to the G-quadruplex, on binding to Gq23 under conditions of both Na^+^ and K^+^ was evaluated. The CD spectra for proteins and the spectra for G-quadruplex DNA do not overlap; so the effect of protein binding on the DNA structure could be observed. For these experiments, spectra were recorded at increasing molar ratios of RPA or RPA-CDE-core to Gq23. In the presence of Na^+^, both RPA and RPA-CDE-core efficiently unfolded Gq23 as seen by a decrease in the magnitude of the peak at 292 nm (Figures 4(a) and 4(c), Table 1 (line 1)). For RPA the unfolding was much more efficient and was complete at a molar ratio of 2. This is probably because it contains the primary DBDs-A and -B of RPA1, so the DNA binding footprint is larger, and RPA has an overall higher affinity than RPA-CDE-core. With K^+^ ions however, this unfolding by both RPA and RPA-CDE-core is much less efficient due to the stabilizing effect of K^+^ ions on Gq23. Even at a molar ratio of 5, both RPA and RPA-CDE-core do not completely unfold the G-quadruplex (Figures 4(b) and 4(d), Table 1 (line 2)). Note that the antiparallel peak (at 292 nm) was more susceptible to unfolding by RPA than the parallel one (at 260 nm).

### 3.5. Stabilizing Effects of G-Quadruplex Ligands

**Figure 5:**
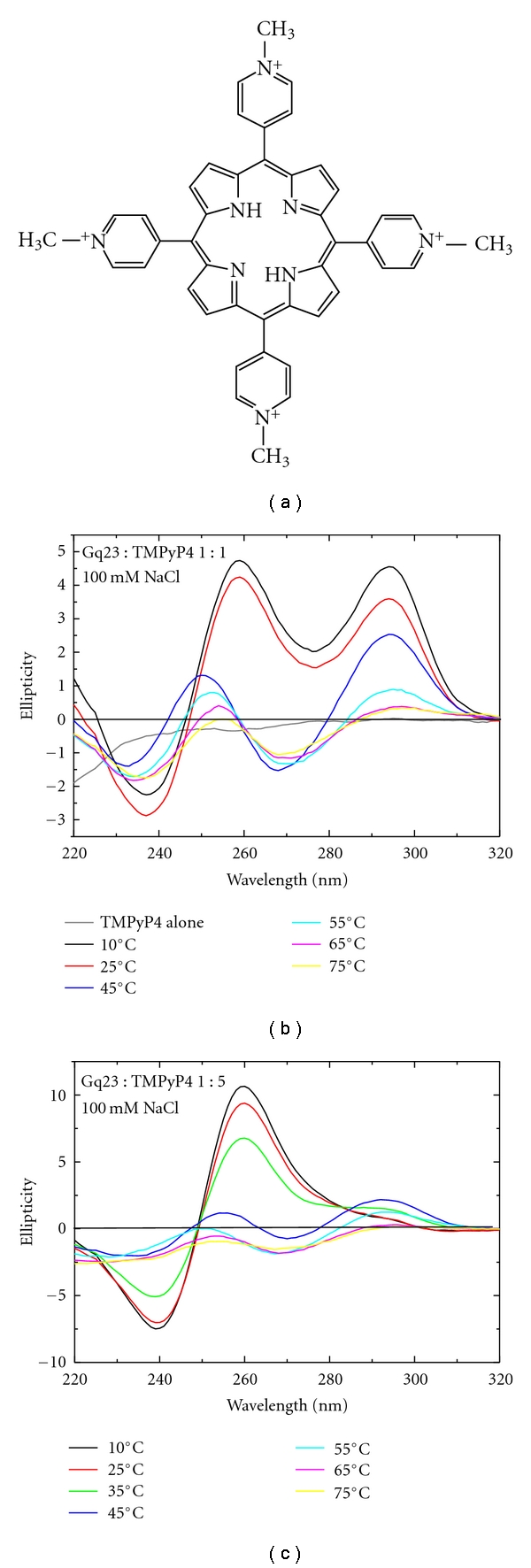
Effect of TMPyP4 on Gq23 in Na^+^ ions. (a) Chemical structure of the porphyrin TMPyP4. TMPyP4 was combined with Gq23 in a 1 : 1 (b) or 1 : 5 (c) molar ratio of Gq23 : TMPyP4.

**Figure 6:**
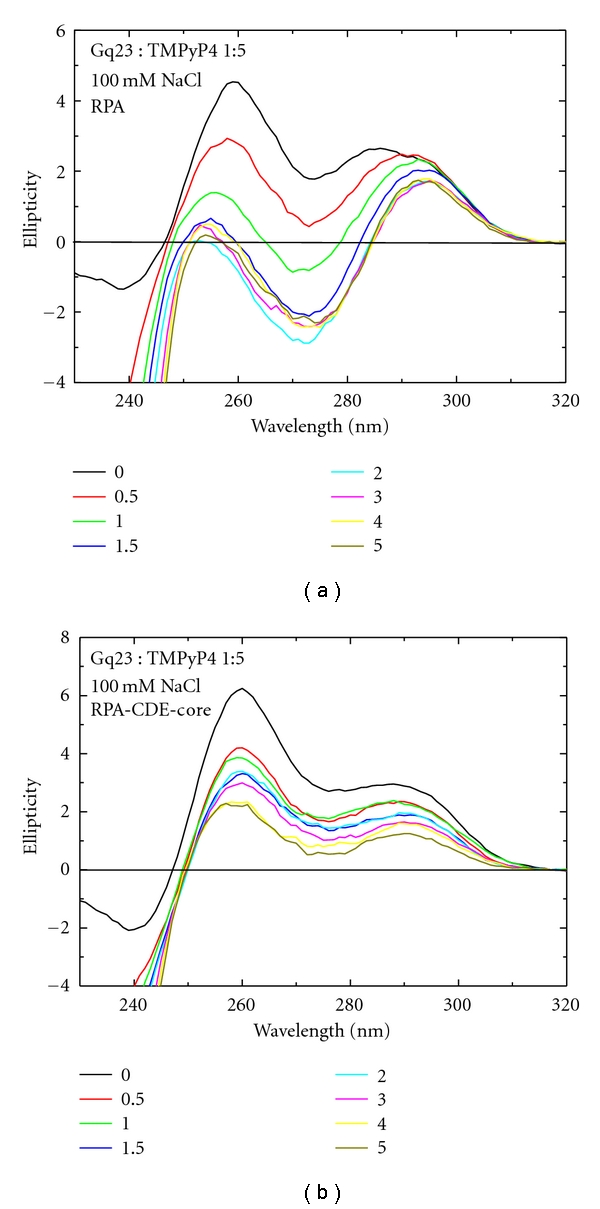
Effect of TMPyP4 and RPA on Gq23 in the presence of Na^+^ ions. (a) CD spectra of Gq23 at a 1 : 5 molar ratio of Gq23 : TMPyP4 (black line). Spectra were collected upon addition of increasing molar ratios (0–5) of RPA or (b) RPA-CDE-core. All spectra were recorded in a buffer containing 100 mM NaCl.

G-quadruplex stabilizing agents such as porphyrins and telomestatin have been successfully used to stabilize G-quadruplex structures. In order to analyze the effect of porphyrins on Gq23, binding to the widely used TMPyP4 molecule (Figure 5(a)) was studied. TMPyP4 caused interconversion of the antiparallel G-quadruplex at 292 nm to the parallel form at 260 nm (Figures 5(b) and 5(c), black line, compared to Figure 1(a) red line). Only partial interconversion was observed at the 1 : 1 ratio (Figure 5(a)) but almost complete interconversion was observed at the 1 : 5 ratio (Figure 5(b)). This type of interconversion between G-quadruplex structural types by porphyrin binding has been observed previously [46]. TMPyP4 alone did not have a CD signal in these buffer conditions (data not shown). Gq23 : TMPyP4 (1 : 1) in 100 mM NaCl shows a slight blue shift of the 260  nm CD peak, at 45°C, and shifts further to the blue wavelengths at higher temperature (Figure 5(b)). TMPyP4 did not thermally stabilize Gq23 in Na^+^ (Table 1 (line 3)). Addition of RPA caused the peak at 260 nm to unfold completely (Figure 6(a)). The addition of RPA-CDE-core also caused the peak at 260 nm to diminish although complete unfolding was not seen even at a molar ratio of 5 (Figure 6(b), Table 1 (line 3)). This indicates a small stabilizing effect of TMPyP4 in Na^+^ (compare Figures 4(a) and 4(c) with Figures 6(a) and 6(b)). 

**Figure 7:**
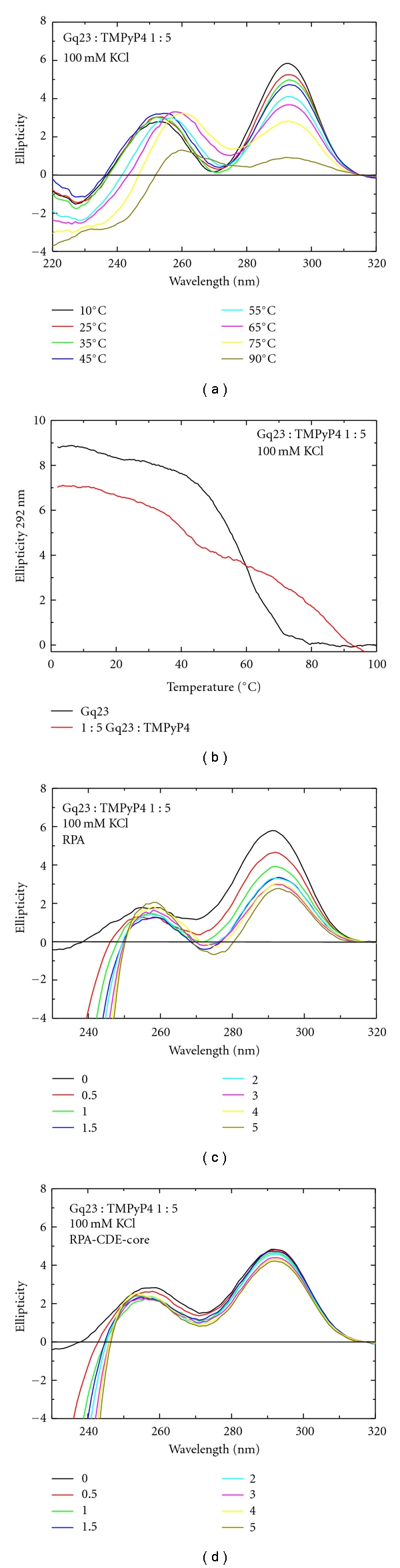
Effect of TMPyP4 and RPA on Gq23 in the presence of K^+^ ions. (a) CD spectra of Gq23 at varying temperatures in the presence of a 1 : 5 molar ratio of Gq23 : TMPyP4. (b) Melt at 292 nm indicates thermal stabilization by TMPyP4 in 100 mM KCl. (c) CD spectra of Gq23 at a 1 : 5 molar ratio of Gq23 : TMPyP4 (black line). Spectra were collected upon addition of increasing molar ratios (0–5) of RPA or (d) RPA-CDE-core. All spectra were recorded in a buffer containing 100 mM KCl.

Since Gq23 formed a more thermally stable G-quadruplex structure in the presence of KCl, the effect of TMPyP4 binding was studied in a buffer containing 100 mM KCl. Increasing the temperature to 90°C indicated that the G-quadruplex was more stable in the presence of TMPyP4 under conditions containing K^+^ (Figure 7(a)). A melting curve monitored at 292 nm revealed that the presence of TMPyP4 caused a biphasic transition with *T_M_* values at 40°C and 80°C. The latter value presents a >20°C increase from the *T_M_* in the absence of TMPyP4, indicative of substantial thermal stabilization (Table 1 (lines 2 and 4)). The biphasic nature of the melting corresponds with the notion of 2 porphyrins binding to the G-quadruplex. TMPyP4 preferentially binds parallel (10^7^) over antiparallel (10^6^) G-quadruplexes [47]. In our bimodal melting curve, the end stacking interaction is thought to bind more tightly (10^7^) than the external binding (10^6^); so it would come off last [47].

Protein binding to Gq23 in the presence of TMPyP4 under conditions containing K^+^ was studied. It was noted that increasing amounts of RPA caused the peak at 292 nm to diminish slightly but complete unfolding was not observed even at a molar ratio of protein : Gq23 of 5 : 1 (Figure 7(c) compared with Figure 4(b)). When RPA-CDE-core was titrated into the Gq23 : TMPyP4 complex, no unfolding was seen to occur (Figure 7(d), Table 1 (line 4)), indicating either that this Gq23 : TMPyP4 complex is very stable in the presence of K^+^ or that protein binding to the complex is inhibited. 

**Figure 8:**
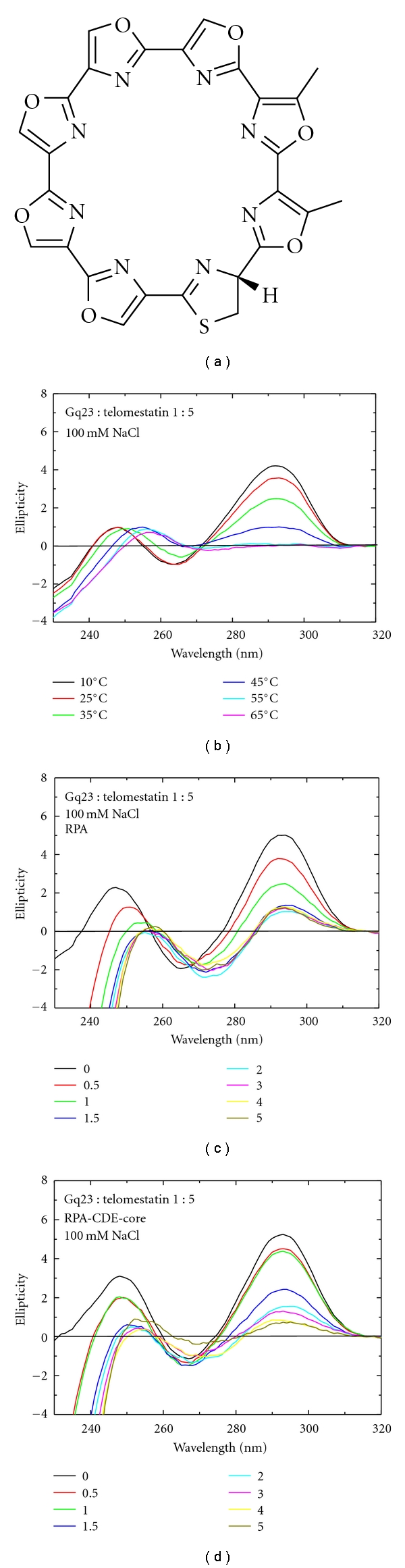
Effects of telomestatin and RPA on Gq23 in the presence of Na^+^ ions. (a) Chemical structure of telomestatin. (b) CD spectra of Gq23 at varying temperatures in the presence of a 1 : 5 molar ratio of Gq23 : telomestatin. (d) Spectra of increasing molar ratios of RPA (0–5) added to above Gq23 : telomestatin complex and (d) spectra of increasing molar ratios of RPA-CDE-core added to the Gq23 : telomestatin complex. All spectra were recorded in 100 mM NaCl.

The binding of telomestatin, another molecule that specifically binds G-quadruplex structures, was also studied (Figure 8(a)). In the presence of Na^+^ ions, at a 1 : 5 ratio of Gq23 : telomestatin, interconversion to the parallel form was not observed (Figure 8(b)) and telomestatin did not alter the *T_M_* of Gq23 under these conditions. To address whether Gq23 stabilization by telomestatin was sufficient to prevent unfolding of the oligonucleotide by RPA or RPA-CDE, a titration experiment was performed. A 1 : 5 complex of Gq23 : telomestatin was preformed and increasing molar ratios (0.5 to 5) of RPA (Figure 8(c)) and RPA-CDE-core (Figure 8(d)) were added. Even at low molar ratios of protein : ssDNA, both proteins readily unfolded the G-quadruplex. Complete unfolding occurred at a ratio of 1.5 for RPA and 3 for RPA-CDE-core as seen by the decrease in the peak at 292 nm. This indicates that telomestatin does not inhibit the unfolding of the G-quadruplex by either RPA or RPA-CDE-core. 

**Figure 9:**
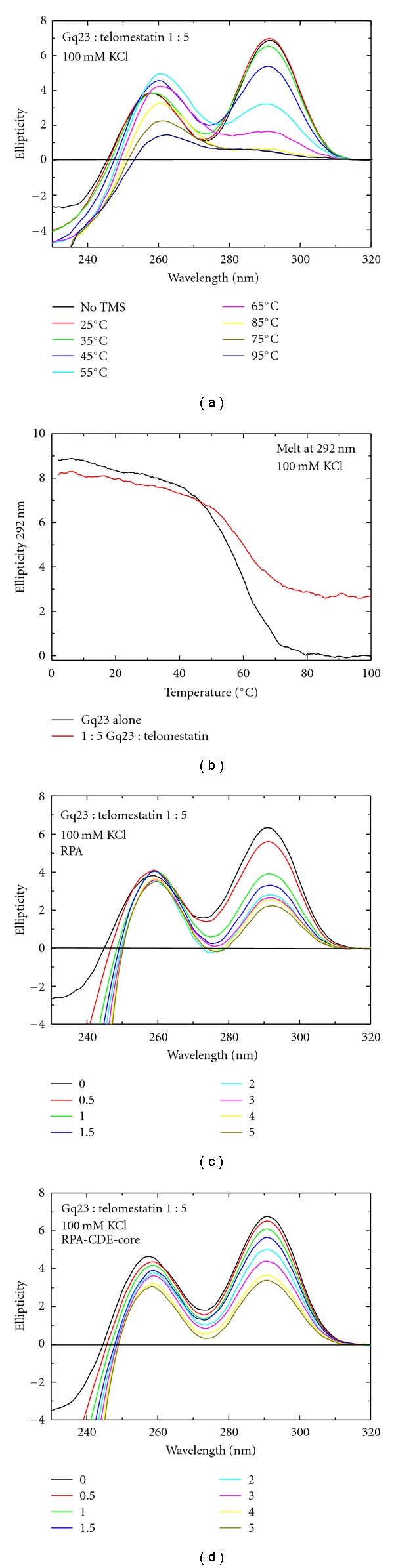
Effects of telomestatin and RPA on Gq23 with K^+^ ions. (a) Gq23 : telomestatin 1 : 5 at increasing molar ratios. (b) Melt at 292 nm of Gq23 alone (black) and 1 : 5 Gq23 : telomestatin (red) indicates no thermal stabilization of Gq23 upon addition of telomestatin. (c) Spectra of increasing molar ratios of RPA (0–5) added to above Gq23 : telomestatin complex and (d) spectra of increasing molar ratios of RPA-CDE-core added to the Gq23 : telomestatin complex. All spectra were recorded in 100 mM KCl.

A similar experiment with telomestatin was performed in the presence of K^+^. At a 1 : 5 molar ratio of Gq23 : telomestatin, no thermal stabilization was observed (Figure 9(a)). Using a melting curve, the *T_M_* values obtained with and without telomestatin were similar (Figure 9(b), Table 1 (line 6)). Spectra of the titration of proteins RPA and RPA-CDE-core (Figures 9(c) and 9(d)) were very similar to those obtained without telomestatin (as seen in Figure 4). These results indicate that telomestatin does not have a significant stabilizing effect on Gq23 in the presence of either Na^+^ or K^+^. Also, telomestatin apparently has no effect on protein binding.

## 4. Discussion

G-quadruplex ligands have been studied extensively in regard to their binding to and effect on G-quadruplex structures. Methods include biochemical assays that study the inhibition of polymerase (PCR-stop) and inhibition of telomerase (TRAP-G4, telomere repeat amplification protocol) activities as well as dimethylsulfate (DMS) footprinting and electrophoretic mobility shift assays (EMSAs) that detect the folding of G-quadruplexes upon ligand binding [48]. Several studies have used CD to measure the effects of ligand binding on G-quadruplex structure. The studies presented here take the use of CD a step further and use it to directly measure the effect on the G-quadruplex fold by RPA binding and to assess the effect of ligand binding to G-quadruplex unfolding by RPA. 

The overall purpose of this study was to find conditions where a small-molecule G-quadruplex ligand inhibited the unfolding of the intramolecular G-quadruplex Gq23 that binds selectively to RPA [38]. Here, the unfolding of Gq23 by RPA and RPA-CDE-core under conditions containing either K^+^ or Na^+^ and in the presence of G-quadruplex ligands was studied. Using CD techniques, it was determined that Gq23 formed a more thermally stable G-quadruplex in a buffer containing K^+^ ions. The addition of the porphyrin TMPyP4 did not alter the thermal stability of the G-quadruplex in the presence of Na^+^ but caused a significant stabilization under conditions containing K^+^ ions. The K^+^/TMPyP4-stabilized form of the G-quadruplex could not be completely unfolded by RPA. 

Not only do G-quadruplex ligands selectively bind G-quadruplex structures over double-stranded DNA (dsDNA) but they also seem to differ in their selectivity for G-quadruplex sequences and folds [15, 49]. For example, telomestatin had 70-fold selectivity for intramolecular telomeric G-quadruplexes over dsDNA when studied by EMSA. In the same study, TMPyP4 converted the same telomeric oligonucleotide into an intramolecular G-quadruplex structure. Telomestatin and TMPyP4 were equipotent at stabilizing the c-Myc and telomeric intramolecular G-quadruplexes as studied using TRAP-G4 and PCR stop assays. Unfortunately this “stabilization” was not studied by CD and *T_M_*'s were not measured; so a direct comparison is not possible. It is known that the binding of telomestatin converts preformed mixed parallel/antiparallel intramolecular telomeric G-quadruplexes to the antiparallel basket form in the presence of K^+^ [50, 51]. The binding of telomestatin on Gq23 had no effect on the CD spectra, but TMPyP4 did. As telomestatin is active at much lower concentrations than TMPyP4, this difference is significant. Interestingly, the G-quadruplex sequence studied here, Gq23, more closely resembles a G-rich promoter sequence, in particular the 3′ end of the hTERT core promoter G-rich region [9].

The differential effects of G-quadruplex ligand binding to G-quadruplex forming sequences have been studied and the results varied depending on the sequence used. In a study using FRET, telomestatin increased the *T_M_* of a 21-mer telomeric G-quadruplex by ~24°C in the presence of Na^+^ or K^+^ ions. TMPyP4 increased the *T_M_* by 21°C in Na^+^ and 27°C in K^+^[52]. It is noteworthy that TMPyP4 does not always increase the *T_M_* of G-quadruplexes. For example, when stabilization of the mixed parallel/antiparallel *BCL-2* promoter G-quadruplex was studied by UV thermal melts, the change in *T_M_* was only 2°C [53]. The PDGF-A promoter has two major intramolecular, parallel G-quadruplex structures on the 3′ and 5′ end that were differentially stabilized by telomestatin (3′) and TMPyP4 (5′), as shown by the polymerase stop assay [54]. TMPyP4 also specifically inhibited PDGF-A proximal promoter activity in vivo. The binding of telomestatin did not change the CD spectra of the parallel intramolecular RET promoter G-quadruplex [55] although it did inhibit the polymerase stop assay. Thus the differential effects of TMPyP4 and telomestatin on these promoter G-quadruplex forming sequences are complex and not currently well understood. 

## 5. Conclusions

G-quadruplex ligands can be envisioned to have at least three potential uses: to inhibit telomerase, to inhibit transcription, and to inhibit DNA replication. Note that for the latter to occur with fidelity, RPA must be able to bind and unfold the G-quadruplexes, so as to minimize the possibility of harmful deletions occurring. In fact, expression of a mutant RPA in human cancer cells caused telomere shortening [56] suggesting that RPA is needed for telomere-length regulation. Also, RPA is thought to have a telomere capping function in ALT tumor cells [57]. Telomestatin is known to inhibit telomerase but does not inhibit DNA replication [58]. Although telomestatin does not inhibit DNA replication in cells, it does block polymerase activity in vitro [15]. So maybe other factors, like RPA, are at work in cells and allow DNA replication in the presence of telomestatin. This would lower the toxicity of telomestatin. In general, the literature reveals a dearth of knowledge when it comes to observing the thermal stabilization effects of telomestatin on G-quadruplexes. In our study, since a stabilization effect was observed with TMPyP4, it is possible that molecules like this porphyrin could be used to stabilize G-quadruplex DNA in vivo to block replication or introduce errors during DNA replication in fast dividing cells such as cancer cells. This could negatively affect the replication of G-rich DNA with severe consequences for the cell such as cell senescence, telomere shortening, and even apoptosis. TMPyP4 porphyrin is known to have a high affinity for G-quadruplex DNA, but with a low selectivity [59]. This low selectivity is the likely cause of the cytotoxicity observed in studies employing human cell lines. Thus TMPyP4 is currently only useful as an in vitro tool rather than a chemotherapeutic. Interestingly, new porphyrins such as diseleno sappyrin show dramatically improved selectivity, and research groups are actively working to modify porphyrins to have better pharmaceutical properties.

## Figures and Tables

**Table 1:** Spectroscopic melting parameters and RPA unfolding of Gq23 in various conditions.

Sample conditions		260 nm		292 nm		RPA^a^	RPA-CDE^a^-core
	*T*_*M*_^b°^ C	*T_M_*°C	Δ*H*_VH_ kcal	*T_M_*°C	Δ*H*_VH_ kcal		
(1) Na^+^				40	23	++	++
(2) K^+^		66	32	57	35	+	+
(3) TMPyP4 + Na^+^	<45					+	+
(4) TMPyP4 + K^+^				40/80^c^	78/58	+	−
(5) Telomestatin + Na^+^	<45					++	++
(6) Telomestatin + K^+^				60	38	+	+

^a^++ complete unfolding, + partial unfolding, − no unfolding detected.

^b^Estimated from their CD spectra at several temperatures.

^c^Melting curve was biphasic.

## Abbreviations

CD: Circular dichroism
DTT: Dithiothretiol
EMSA: electromobility shift assay
FP: Fluorescence polarization
FRET: Fluorescence resonance energy transfer
OB-fold: Oligonucleotide-binding fold
RPA: Replication protein A
SELEX: Systematic evolution of ligands by exponential enrichment
ssDNA: single-stranded DNA.
